# Supplementary material for: Patient Perspectives on Social and Identity Factors Affecting Multiple Myeloma Care: Barriers and Opportunities
Source: Healthcare (Basel). 2024 Aug 9;12(16):1587. doi: 10.3390/healthcare12161587 (PMC11354118; doi:10.3390/healthcare12161587)
Supplement: Supplementary file 1 [file healthcare-12-01587-s001.zip › healthcare-3105503-supplementary.pdf]

Focus Group 1 Moderator's Guide: Key Questions and Overall Agenda for a 2-hour Discussion

- Welcome & Ground Rules (5 min)
- Introductions (5 min)
- Better Understand Multiple Myeloma and How it Differs from Other Chronic Health Conditions (25 min)
    - Based on our last conversation, PERC members identified three main themes related to living with Multiple Myeloma:
        - Ongoing anxiety
        - Frequent side effects
        - Limited initial information on Multiple Myeloma and treatment options
    - What is your reaction? Do these resonate with you? Please tell us more about how these are characteristic of multiple myeloma.
    - PERC members saw MM as unique related to:
        - How and where diagnosis is made
        - Disease progression and relapse
        - Treatments and side effects
        - Financial issues, including cost and coverage
        - Toll on physical and emotional health
        - Appointment frequency and length of stays
    - What is your reaction? Do these resonate with you? Please tell us more about how MM is different in these areas.
- Impact of Social Experiences (30 min)
    - PERC members identified the following social experiences as negatively impacting their care journey:
        - Limited health knowledge
        - Lack of insurance coverage
        - Financial instability
        - Lack of transportation
        - Lack of social support
    - Quantification exercise: At which point(s) along the care journey do you think <u>social experience</u> has significant negative impacts? Please select all that apply.
        - Diagnosis
        - Treatment goals
        - Treatment choice

        - Treatment initiation
        - Treatment adherence
        - Burden of disease/disease outcomes
        - None of the above
    - Discuss results
- Break (5 min)
- Impact of Identity Experiences (25 min)
    - PERC members identified the following identity experiences as negatively impacting their care journey:
        - Race/ethnicity
        - Age
        - Culture
        - Disability status
    - Quantification exercise: At which point(s) along the care journey do you think identity experience has significant negative impacts? Please select all that apply.
        - Diagnosis
        - Treatment goals
        - Treatment choice
        - Treatment initiation
        - Treatment adherence
        - Burden of disease/disease outcomes
        - None of the above
    - Discuss results
- Opportunities and Solutions (20 min)
    - Where along the care journey is support most needed to address the impact of social and identity issues? What might help?
    - Exercise: As a group, allocate 100 points to distribute along the care journey, with more points going toward areas where support would be most impactful. Discuss results.
- Closing (5 min)

Focus Group 2 Moderator's Guide: Key Questions and Overall Agenda for a 2-Hour Discussion

- Welcome & Ground Rules (5 min)
- Introductions (5 min)
- Support at Different Stages: Diagnosis (40 min)
    - What support would be most helpful for patients at the time of diagnosis? Think about support related to:
        - Basic disease information
        - What it means to live with MM (e.g., likely impacts on day-to-day life and long-term plans)
        - Treatment options
        - Access to specialists
        - Financial assistance
        - Transportation or logistical support
        - Emotional support
    - What examples do you have of helpful support you received at the time of diagnosis?
    - Think about what kind of support would be most helpful for patients at the time of diagnosis. Consider:
        - What it looks like
        - Who it involves (e.g., providers, patient advocates, social workers, psychologists, insurance, etc.)
        - How it is provided
        - Touchpoints/frequency
- Break (5 min)
- Support at Different Stages: Adherence (30 min)
    - What support would be most helpful for patients to support adherence? Think about support related to:
        - How treatments work
        - Why adherence is important
        - Managing complexity of treatment plan
        - Coping with side effects
        - Financial assistance
        - Navigating insurance
        - Transportation or logistical support

        - Emotional support
    - What examples do you have of helpful support you received related to adherence?
    - Think about what kind of support would be most helpful for patients related to adherence. Consider:
        - What it looks like
        - Who it involves (e.g., providers, patient advocates, social workers, psychologists, insurance, etc.)
        - How it is provided
        - Touchpoints/frequency
- Support at Different Stages: Treatment Choice (30 min)
    - What support would be most helpful for patients related to treatment choice? Think about support related to:
        - How treatments work
        - Personalized information about treatment efficacy
        - Logistical information related to treatment choice (e.g., cost, coverage, transportation, etc.)
        - How to make a selection among treatments
        - Preparing for next treatment
        - Future treatments which are being developed
    - What examples do you have of helpful support you received related to treatment choice?
    - Think about what kind of support would be most helpful for patients related to treatment choice. Consider:
        - What it looks like
        - Who it involves (e.g., providers, patient advocates, social workers, psychologists, insurance, etc.)
        - How it is provided
        - Touchpoints/frequency
- Closing (5 min)
